# Verbal Communication with the Patient Is Not Enough: The Six Languages of the Sick

**DOI:** 10.3390/nursrep12040072

**Published:** 2022-10-13

**Authors:** Carlo Valerio Bellieni

**Affiliations:** 1 Department of Molecular Medicine and Development, University of Siena, 53100 Siena, Italy; bellieni@unisi.it; 2 Bioethics Committee, Tuscany Region, 50100 Florence, Italy

**Keywords:** communication, palliative care, holistic care, pain, language

## Abstract

Evidence shows that verbal communication is just one of the ways patients indicate their wishes. For a sufficiently careful communication, we should also grasp other five unusual though evident languages: (a) body language, (b) the way patients manage their environment, (c) unconscious language, (d) lab-evidenced language, and (e) the way they master technology. So, we have six languages that should be intertwined to understand the real language of the sick. Grasping these languages helps health professionals frame the patient’s mood, their level of suffering or mental growth, and understand what words alone cannot express. Words cannot express completely what a patient senses: for subjection, shyness, because some patients are still non-verbal or because verbal communication is just a useful way of freezing concept but has not the same fluidity and liberty of the other above-described languages. It is mandatory for caregivers to wonder how many of these languages they are actually decrypting during an interview with the patient. On the other hand, caregivers unconsciously communicate much through two unexpected languages: the architectural language and the language of medical procedures. The way they welcome or obstruct the patient, their hesitations across a treatment, or in showing a serene collegiality are forms of subtle communication. A paradigmatic scenario where all these languages should be implemented is the “informed consent” process, which should be turned into a “shared therapeutic pathway”, summing up all the communicative modes illustrated in the text.

## 1. Communication with Sick Patients and Their Families

### 1.1. The Binary Communication

Current healthcare is based on binary communication: you must ask a net question and the patient must answer with a net utterance. This is not communication; this is computer language. Human communication is multifaceted and holistic; binary communication can give you at most the answer you are looking for, but omits many other details, sometimes important or even determinant for a diagnosis. This is basic for seriously ill patients, most of whom are non-verbal and often admitted to palliative care wards.

### 1.2. Communication in the Palliative Care Setting

The World Health Organization recently underlined the importance of communication in the palliative care setting [1] (Table 1): “Early identification of palliative care needs by primary care providers has been found to depend on:

- Clinicians’ knowledge, skills and communication styles
- Patients’ communication styles;
- Quality of the clinician–patient relationship;
- Patient’s perceptions of the clinician’s role;
- Level of collaboration between the primary care clinician and other clinicians;
- Patient’s fears and beliefs about the prognosis.” [2]

In 2020, a Consensus Conference on Palliative Care [3] pointed out that pain must no longer be “palliated”, that is, covered, but must come to light in all its expression and expressiveness. Even for babies, patients urge clinicians to use a “holistic-evolutive approach to pediatric palliative care [4]. The new holistic approach to palliative care indicates that it is neither limited to those who know how to claim their treatment, nor to those who have a simple disease, well described in words, as if care were only for those who deserve it. 

Even the treatment of pain—a central point of palliative care—has had an important evolution in recent years under the communicative perspective: while the previous definition of the word pain [5] did not include those who were unable to describe their painful sensation in words, in 2020, the International Association for Studies on Pain introduced a new definition [6] that also includes those who do not know or cannot express themselves in words (Table 2). 

I recently performed a thorough analysis of communication in palliative care [4], and I found out that the patient–caregiver communication is multifaceted. It is a challenging field to help both in performing optimal healthcare (Table 3). To this aim, I have reviewed the modern principles of semiology and those papers that describe the patients’ needs, with particular correlation with palliative care. 

### 1.3. Multilanguage

Modern semiology has shown that speech is just one of the possible languages that humans use. One of these are gestures: “Unlike language signs, gestures are characterized by highly variable semiotic profiles that are shaped in multimodal usage events, and they form a fluid system with unique qualities.” [7]. Nonetheless, human communication passes through other channels, as well. 

People use multi-language communication, that is, it is not limited to verbal language, and being cared for should neither be the prerogative of those with a pathology describable in a few words, nor of those who know how to express themselves: “Verbal description is only one of several behaviors to express pain”, the IASP added in its definition of pain [6]. The philosopher Judith Holler recently wrote: “The human communication system that we consider to be the explanandum in the evolution of language thus is not spoken language. It is, instead, a deeply multimodal, multi-layered, multifunctional system that developed-and survived-owing to the extraordinary flexibility and adaptability that it endows us with” [8]. Palliative care should use a multi-language approach because most of their patients are non-verbal or are so prostrated that speech is the least important of their communication tools. Much is not transmitted in words: even the unconscious is language [9].

## 2. The Six Languages of the Sick Patient

**Figure 1:**
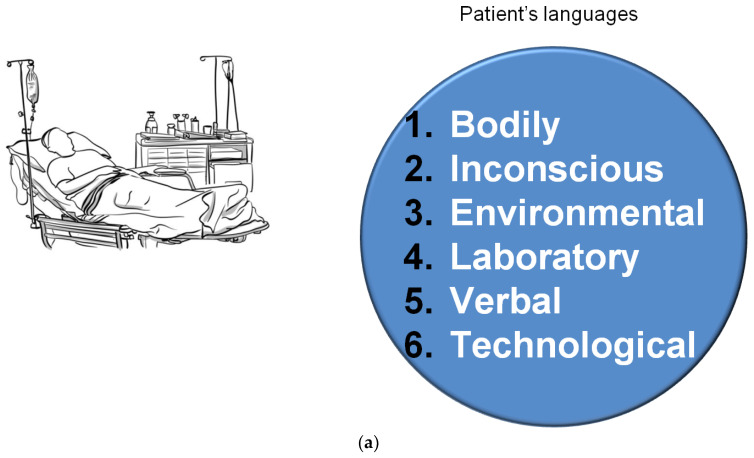

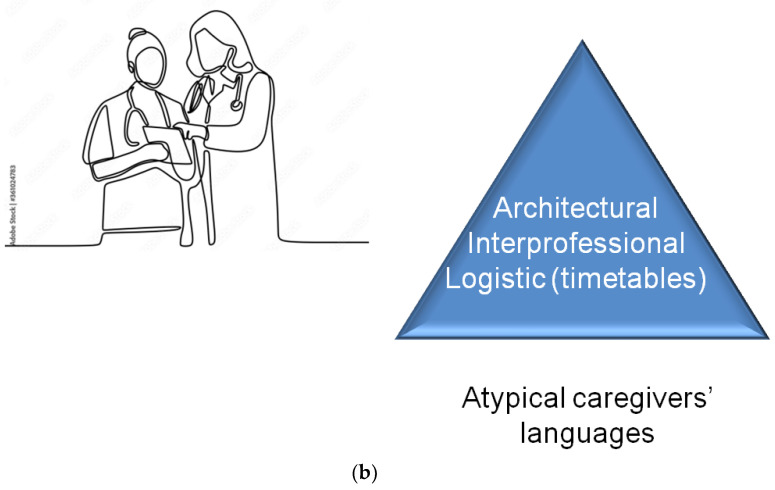
(**a**) Patients’ multiple languages. (**b**) Three atypical ways of communication.

The analysis of the literature on the behavior of hospitalized patients and on semiology points out that at least six languages are used by the patients. These languages are intertwined with each other, any one reveals details of the others. Because humans are not unidimensional, in particular, hospitalized patients add to their voice’s power other expressive modes (Figure 1a). Words lose their power for a sense of subjection, for weakness, for being naturally non-verbal in some cases, or for having to express decisions or ideas that they cannot express in words. 

**Environmental language.** It is the way patients change or restore the hospital environment, their personal boundaries, the way they treat their things [10], their hygiene and wearing. Personal disorder, mess, or the need of tidying up has an utter communicative meaning [11]. Mess or order can be considered metonymies, a rhetoric figure of speech that, following Lacan, disclose our unexpressed desires [12].

**Techno language.** The symbol of today’s world is technology and mass media: the way the patient interacts with them tells us how the patient is integrated into their world [13]. The way they use them or how are themselves used, how they ignore them or become their slaves, how they seek them or fear them, are also forms of expression [14].

**Body language.** The silences “speak” [15], as do the attitudes of the body and face: they are a crucial part of language. For some populations, mimic expressions are integral to their language [16]. However, even in industrial societies, where corporeality has lost value due to the progressive mutual estrangement of individuals, mimicry remains very important, especially in cases of anguish or difficult choices [17]. We often see patients saying one thing in words and showing another with their bodily acts. They also highlight concepts with gestures or express them with screams, redness, sudden pallor, with a look that asks for help, biting their lips, or stressing their jaws.

**Biological language.** Through the analysis of body movements and hormone production, we can evaluate the stress level of patients as well as their pain, depression, anxiety [18]. To this purpose, different scales were developed, which measure the events mentioned above by mixing various vital parameters, from oxygen saturation to heart rate, from cortisol production to electrical conductance, to other components [19]. This is a way to give voice to the voiceless. On this basis, the pain principle was created [20]: it is a criterion for modulating intensive care based on the level of stress objectively detected in those who cannot express themselves in words.

**Unconscious language.** Lapses, humorous jokes, repetitions of concepts and forgetfulness are often signs of an unconscious activity [21]. The unconscious is not a monster fighting us from within, but it is a way our psyche speaks. It must come out because, as Lacan said, “it speaks where it hurts” [22], that is, our psyche expresses its discomfort in analogy with the cause that provoked it [23]. Sometimes this will be evident, as in those cases of anorexia due to a rejection of life. Even pictures and drawioften ng made by patients, in particular children, and hastily considered as “naivety “can express more than what they express in words.

**Verbal language.** It is the best known but it is neither the most direct nor the one that necessarily expresses honestly the patient’s thought; in any case, it is not the only language to do it. Verbal language has risen to be considered the dominant language under the influence of positivist philosophy which wanted to see that “all that is real is rational, and all that is rational is real” (Hegel) [24], or, according to Ludwig Wittgenstein, “what cannot be talked about must be kept silent” [25]. This led to the identification of thought with words. The consequence was that people’s thoughts were considered limited to their rationality and their rational language, but rational language, as psychoanalysis revealed, is not necessarily what expresses the patient’s desire. Limiting communication to verbal language also excludes and censors any possibility of conversation and expression of those who do not speak: babies, disabled people, depressed or autistic people, people in a coma, and the mute. Spoken language is so prominent in common use also because of people’s laziness: it is easier to recognize only one language rather than facing the difficulties of multi-language expression. Spoken language, however, although far from being a free and rational tool, has its important function: it conveys an immediate meaning that urges us to interpret it.

## 3. How to Use These Languages

These six languages are the fundament of a holistic–evolutive approach to palliative care [26]. We can use verbal and non-verbal semantic expressions to acknowledge (a) the whole spectrum of human dimensions of the patient, (b) the step where they are across their grief or their growth, and (c) patients’ wishes and needs. This happens through the following:

- Recognizing the patient’s character. In this, we are supported by the studies on the types of characters of Jung and the Myers-Briggs [27].
- Acknowledging the level of development of a minor, and knowing how to interact with him/her: every age has its own conceptualization [28]: from playset [29] to idealizations, from the development of the theory of mind, to magical fantasies [30].
- Recognizing the step of mourning that the patient is going through. Useful in this regard are the well-known categorizations of mourning [31].
- Assessing the level of expressed and unexpressed suffering.

## 4. Doctors’ and Nurses’ Languages

Even caregivers “speak” through unexpected ways. Two non-verbal languages (Figure 1b) are particularly worthy of attention:

- Architectural language.
- The language of medical treatments.

**Architectural language**. The way we structure work and hospital environments is a language and communication. This concerns the architecture [32], the spaces, the views, and the ecological green spaces. What the patient can see or cannot, the spaces he/she has, and the availability of services particularly for motility disabled people are languages: they say overtly whether a patient is welcomed or is reclused. 

**The language of medical treatments**. The therapies are perceived by the patient as a form of language, a sort of communication from the caregivers: routine, novelty, hope, and despair can sometimes be communicated by foregoing, introducing, proposing, withdrawing, or hesitating about a drug or a treatment, without the need for words [33]. This is also the case for the collaboration of their caregivers with other doctors or nurses, that the patient perceives. A serene inter-professional behavior is more reassuring than many words. Even the choice of a medical technical language or a more colloquial one is a way of communicating: if a doctor uses deliberately scientific, jargon or cold terms, either he/she is trying to hide some truth, or he/she does not have clear ideas.

## 5. Informed Consents

A specific scenario is that of the so-called informed consent. The focus on the multiple languages of the hospital setting we have drawn points out that informed consent is much more than what appears on a paper form [34]. If signing a piece of paper was the extent, it would be only a bureaucratic act; however, information is a dialogue, and a dialogue needs a multilanguage. Thus, informed consent should be turned from a formal signature into a process that uses all the possible communicative pathways. Written language simplifies the communication, and this is useful in everyday practice when we give strict information and when we require consent to a procedure. However, it has the disadvantage of crystallizing the words, requiring black/white, yes/no and right/wrong utterances, while real communication necessarily requires shades of grey. Thus, informed consent urges those who propose it to decrypt patients’ true hesitations and wishes, using a multi-language approach.

It is therefore necessary to overcome the rigid concept of “informed consent”, which normally is limited to receiving consent to a series of data provided verbally or in writing. This process is usually doctors’ discharge of responsibility rather than a real involvement of the patient in his/her care (35], despite being born for this latter reason. Three points must make us doubt about this process: (1) the doctor requesting consent is a stranger to the patient; (2) the doctor limits him/herself to illustrating data; and (3) the written text to be signed is redundant and in medical jargon. It is necessary to move from the concept of “informed consent” which is limited to verbal and written language, to “sharing a curative path” which (A) takes care of the patient’s whole history, (B) through a doctor they know and (C) who can grasp most of the languages described above.

## 6. Conclusions

Current healthcare is based on binary communication: you ask a clear question, and the patient answers with a clear utterance. This is what is expected: a yes/no, black/white speech. This is not communication. This is robotics. This happens in the whole body of medical activity.

Healthcare professionals of any branch should learn that their messages are driven by means other than speech and should understand their patients’ multi-language. Otherwise, misdiagnoses are possible, and patients undergo a lack of care. Medicine should unveil suffering and discomfort, managing what or who is not defined with words. It is time that a healthcare system gives a voice to those who have no voice, and interprets the patients’ wishes and fears even beyond their mere words.

As a conclusion, I propose to healthcare professionals an exercise, to contemplate how many of the above six patients’ languages they spot during an interview with a patient. I suggest that a real dialogue is present only when they grasp most of them. 

## Figures and Tables

**Table 1:** Palliative care.

Palliative care is aimed to improve the quality of life of patients and that of their families who are facing challenges associated with life-threatening illness. Each year, an estimated 40 million people are in need of palliative care; 78% of them live in low- and middle-income countries. Worldwide, only about 14% of people who need palliative care currently receive it [1].

**Table 2:** IASP definitions of pain.

1987 definition: “An unpleasant experience that we primarily associate with tissue damage or describe in terms of tissue”
2020 definition: “An unpleasant sensory and emotional experience associated with, or resembling that associated with, actual or potential tissue damage”

**Table 3:** Communication.

The verb “to communicate” is derived from Latin and it means “to bear together a duty” (from the words “cum” that means “with”, and “munus” that means “duty”) such that it is a reciprocal and solidaristic action that requires three standing points:
1. Being companions and not counterparts of those who stand in front of us.
2. Allow a relationship gap to stand between the two: an excessive closeness is negative.
3. Never let those with whom we are communicating think that we are following a protocol and a manual on how to speak with them.
From [4]
